# Genome Sequence of the Piezophilic, Mesophilic Sulfate-Reducing Bacterium *Desulfovibrio indicus* J2^T^

**DOI:** 10.1128/genomeA.00214-16

**Published:** 2016-04-07

**Authors:** Junwei Cao, Lois Maignien, Zongze Shao, Karine Alain, Mohamed Jebbar

**Affiliations:** a Université de Bretagne Occidentale (UBO, UEB), Institut Universitaire Européen de la Mer (IUEM) – UMR 6197, Laboratoire de Microbiologie des Environnements Extrêmes (LMEE), Place Nicolas Copernic, Plouzané, France; b CNRS, IUEM–UMR 6197, Laboratoire de Microbiologie des Environnements Extrêmes (LMEE), Place Nicolas Copernic, Plouzané, France; c Ifremer, UMR 6197, Laboratoire de Microbiologie des Environnements Extrêmes (LMEE), Technopôle Pointe du diable, Plouzané, France; d School of Municipal and Environmental Engineering, Harbin Institute of Technology, Harbin, China; e State Key Laboratory Breeding Base of Marine Genetic Resources, Key Laboratory of Marine Genetic Resources, The Third Institute of State Oceanic Administration, Collaborative Innovation Center of Marine Biological Resources, Key Laboratory of Marine Genetic Resources of Fujian Province, Xiamen, China

## Abstract

The complete genome sequence of *Desulfovibrio indicus* J2^T^, a member of the family *Desulfovibrionaceae*, consists of 3,966,573-bp in one contig and encodes 3,461 predicted genes, 5 noncoding RNAs, 3 rRNAs operons, and 52 tRNA-encoding genes. The genome is consistent with a heterotrophic, anaerobic lifestyle including the sulfate reduction pathway.

## GENOME ANNOUNCEMENT

Sulfate-reducing prokaryotes are those bacteria and archaea that are key players in sulfur cycle on Earth; they can obtain energy by oxidizing organic compounds or molecular hydrogen (H_2_) while reducing sulfate (SO_4_^2−^) to hydrogen sulfide (H_2_S).

*Desulfovibrio indicus* J2^T^ was isolated from a deep-sea serpentinized peridotite sample collected at a depth of 3173 m in a hydrothermal vent area of the Indian Ocean (27°88′ S, 63°53′ E; site 30I-TVG05) (1). *D. indicus*, with *Desulfovibrio hydrothermalis* (2) are the only known sulfate-reducing bacteria (SRB) isolated from deep sea hydrothermal area samples. *D. indicus* is meso-piezophilic growing optimally at 10 MPa (range 0 to 30 MPa). This anaerobic, motile vibrio can use lactate, malate, pyruvate, formate, and hydrogen as energy sources when using sulfate, thiosulfate, sulfite, fumarate, and nitrate as terminal electron acceptors.

Genomic DNA was extracted with the QIAGEN genomic-tip 20/G (QIAGEN, Düsseldorf, Germany) kit following the manufacturer’s standard protocol. Whole-genome shotgun sequencing was carried out using PacBio (Pacific Biosciences, Menlo Park, CA) single-molecule-real-time (SMRT) sequencing technology (Duke University) (3). The genome was sequenced with two PacBio SMRT cells. For the genome assembly, we used the HGAP assembler included in a local installation of the PacBio SMRT portal (V. 2.3.0) with default parameters, This resulted in a single 3,970,855 bp circular genome with a 32× coverage and a G+C content of 63.5%.

Genome was annotated using the NCBI Prokaryotic Genome Annotation Pipeline (http://www.ncbi.nlm.nih.gov/genome/annotation_prok/). A total of 3,461 coding DNA sequences (CDSs) were identified, as well as 90 pseudogenes, 5 noncoding RNAs (ncRNA), 3 rRNAs (5S, 16S, and 23S) operons, and 52 tRNA genes. Additionally, the genome contains one clustered regularly interspaced short palindromic repeat (CRISPR) array associated with seven *cas* (*cas* 1, *cas* 2, *cas* 3, *cas* 4, *cas* 5d, *csd* 1, and *csd* 2/*csh* 2) genes.

Phylogenetic analysis based on 16S rRNA gene sequences showed that strain J2^T^ falls into the genus *Desulfovibrio* within the class *Deltaproteobacteria*, with highest sequence similarity of 98.05% to *Desulfovibrio dechloracetivorans* SF3^T^ (1).

Genes involved in sulfate reduction (4) were identified in the genome, for example, sulfate adenylyltransferase gene (s*at*, AWY79_04190 and AWY79_13965); adenosine phosphosulfate reductase genes (*aprBA*, AWY79_04195 and AWY79_04200); dissimilatory sulfite reductase genes (*dsrAB*, AWY79_17895 and AWY79_17900; *dsrC*, AWY79_11020); sulfate transporter gene (AWY79_06665, AWY79_10840, and AWY79_14480); Genes that mediate the electron transport between the cytoplasmic AprBA and DsrAB and the membrane-integral quinol/quinone pool (4, 5) were also found, for instance, quinone-interacting membrane-bound oxidoreductase genes (*qmoABC*, AWY79_04205 AWY79_04210 and AWY79_04215); sulfite reduction-associated complex protein genes (*dsrMKJOP*, AWY79_04535, AWY79_04540, AWY79_04545, AWY79_04550, and AWY79_04555). The genome also contains a large number of genes encoding hydrogenases, cytochromes *c* and cytochrome *c*-associated membrane redox complexes, which may be possibly involved in electron-transfer and energy conserving pathways (5).

Previous study indicated that energy metabolism of SRB is far more versatile than we considered, so that SRB can use different alternative strategies for energy conservation (5, 6). The genome sequence analysis will allow comprehensive comparisons with other SRB and pave the way for further understanding of SRB lifestyle in anaerobic deep sea environments.

### Nucleotide sequence accession number.

The genome sequence has been deposited in GenBank under the accession no. CP014206.
